# How Does Word Length Evolve in Written Chinese?

**DOI:** 10.1371/journal.pone.0138567

**Published:** 2015-09-18

**Authors:** Heng Chen, Junying Liang, Haitao Liu

**Affiliations:** 1 Center for the Study of Language and Cognition, Zhejiang University, Hangzhou, CN-310028, China; 2 Department of Linguistics, Zhejiang University, Hangzhou, CN-310058, China; 3 Ningbo Institute of Technology, Zhejiang University, Ningbo, CN-315100, China; Stony Brook University, UNITED STATES

## Abstract

We demonstrate a substantial evidence that the word length can be an essential lexical structural feature for word evolution in written Chinese. The data used in this study are diachronic Chinese short narrative texts with a time span of over 2000-years. We show that the increase of word length is an essential regularity in word evolution. On the one hand, word frequency is found to depend on word length, and their relation is in line with the Power law function y = ax^-b^. On the other hand, our deeper analyses show that the increase of word length results in the simplification in characters for balance in written Chinese. Moreover, the correspondence between written and spoken Chinese is discussed. We conclude that the disyllabic trend may account for the increase of word length, and its impacts can be explained in "the principle of least effort".

## Introduction

Word evolution, which basically consists of word formation, word usage and word death, is an essential step for the evolution of human languages [1–5]. It is suggested that word frequency is closely connected to word evolution [6–8]. One case in point is that frequent words tend to be short. This law has been proposed and popularized by Zipf [9,10], suggesting that word frequency is a strong indicator of word evolution. Other than the word frequency, here we put our focus on word length. In fact, there have been an array of studies about word length, for example, Best et al. [11] examined numerous problems concerning word length distributions in many languages including Chinese. However, almost all the previous studies are synchronic ones, and diachronic explorations of one unique language can hardly be seen. In the present study, by exploring the evolution of word length in Chinese written texts, we show that the increase of word length is an essential regularity of word evolution in written Chinese.

The relationship between word length and word frequency has been extensively examined in quantitative investigations of human languages [12,13]. The seminal study in this line is Zipf's grand statement that "the more frequent, the shorter" [9], for which he explained with the "principle of least effort"^10^. Recently a synchronic study about word length and word frequency has yielded outstanding discoveries, which supplies "the largest leap forward in 75 years" in understanding how the evolution of words is governed by the efficiency with which they can be used to communicate: word length is optimized for efficient communication [14,15]. The dependence of word length on frequency has been corroborated by a lot of investigations [9,12,13]. However, prior studies on the dependence of word frequency upon word length are comparatively few. Moreover, as word length evolves over time, its influence on their interrelationships needs to be studied too.

To explore the questions above, we need a manageable number of reliable diachronic texts. Albeit that generally there exists a gap between written and spoken language, which is especially true of Sinitic languages, most of our findings about languages, even in modern times, depend much on written records [16,17]. We reason that Chinese is suitable for our exploration [18,19,20], since it is one of the most archaic living languages for which we have a more or less continuous written record.

In the present study, we use a collection of reliable written Chinese texts ranging from around 300 BC to 2100 AD, which is divided into 6 time periods: BC 3^th^–BC 2^th^, AD 4^th^–AD 5^th^, AD 12^th^–AD 13^th^, AD 16^th^–AD 17^th^, Pre-AD 20^th^, AD 21^th^. The scale of the whole texts collection in each time period ranges from about ten thousand to two million characters. To have a reliable measurement of word length (measured in characters) distributions [11], we have taken two important steps. Firstly, we select a sample of ten thousand-character text from the texts collections in each time period randomly; secondly, we divide the sample of each period into blocks of *N* = 1000, *N* = 2000, *N* = 3000 texts (for *N* = 3000, only 3 blocks are obtained). Then we compute the word length distributions within each block, and obtain the mean word length distribution as well as other statistics over all the blocks.

In the next section **Results**, we quantitatively investigate into a collection of Vernacular Chinese texts spanning more than 2000 years. The evolution of word length distribution, mean word length, the relation between word length and type-token ratio, as well as the relation between mean word length and the exponent *b* in Zipf's law are the key points of interest in this investigation. In **Discussions**, we analyze how word length evolves in written Chinese from a general and dynamic point of view, and endeavor to bring to light why word length increases in written Chinese, as well as the implications. Details of the written texts, methods and statistics used in this paper are displayed in **Materials and Methods** and S1 File.

## Results

### Evolution of static word length distribution

There are two kinds of word length distributions, one is static, which is based on word types, and the other is dynamic, which is based on word tokens. Static word length distribution reflects how a language's lexicon is constructed from its lower units (which are characters in written Chinese), as well as lexical richness of a language.

**Fig 1:**
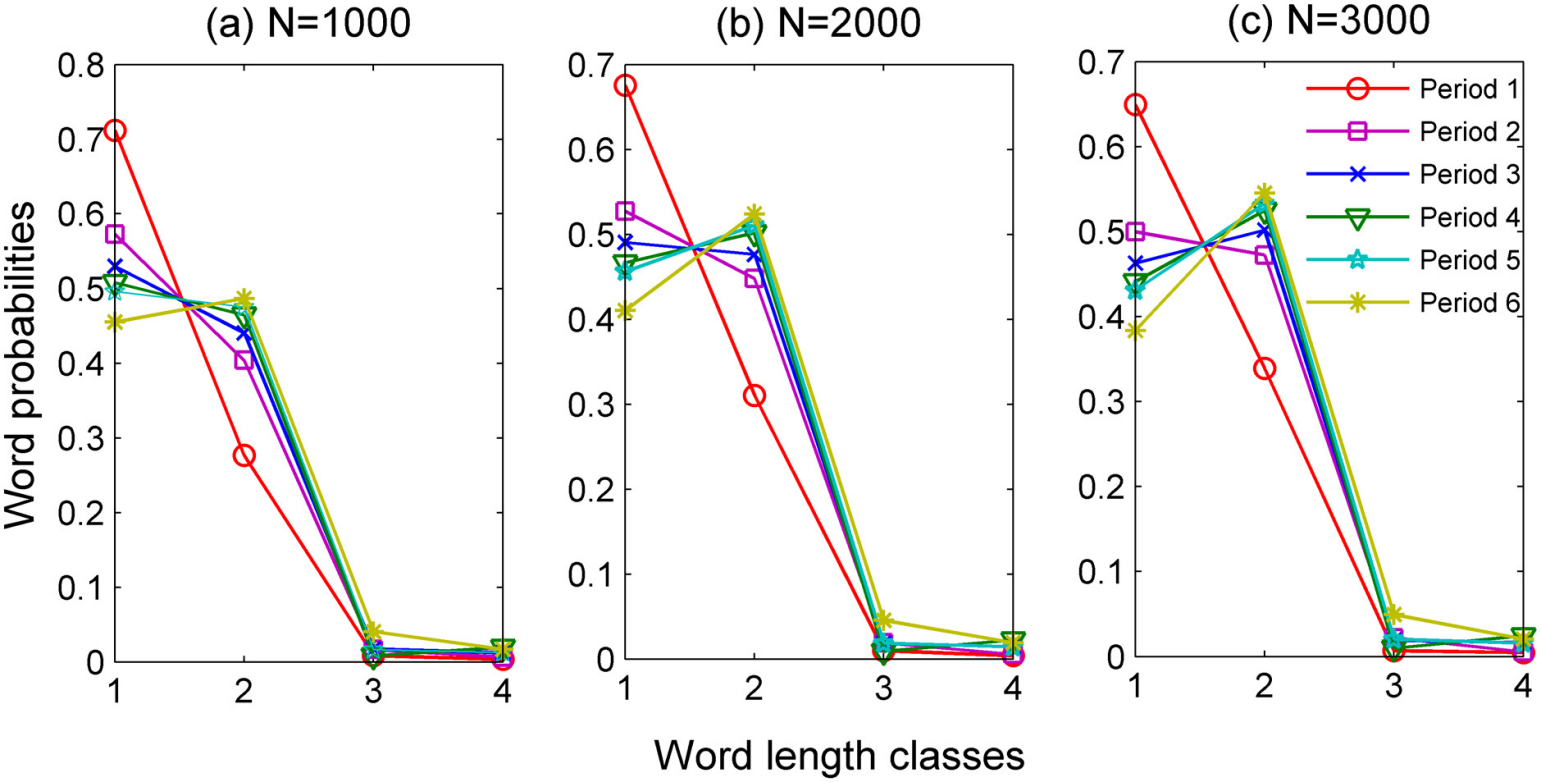
Static Word length distributions of each time period with different texts scales (*N* = 1000, *N* = 2000 and *N* = 3000 characters; statistics for the sample in each time period can be found in S1 File).

Fig 1 shows the diachronic change of static word length distribution of written Chinese (with normalized words probability). We use Word Length Class (hence WLC for short) *x* to represent all those words whose word length equal *x* (measured in characters).

As we can see in Fig 1, based on different texts scales, the static word length distribution probabilities in WLC 1 show a decreasing evolutionary trend, while those of WLC 2, 3 and 4 an increasing evolutionary trend.

As stated above, the shown values are averages over the word probabilities of all segments of the texts of each time period. Meanwhile, to investigate the dependence of the results on segment length *N*, we have repeated the calculations for more segments lengths. It is clear that although word length distributions are sensitive to the choice of scale *N*, the evolutionary trend of each word length class remains unaltered and similar to what we have found for *N* = 1000.

According to previous studies [21–24], from perspective of linguistic typology, the standard static word length distribution should be bell-shaped. As displayed in Fig 1, the pattern of Chinese word length distribution is developing towards the bell-shaped, basically the same as many Indo-European languages today.

**Fig 2:**
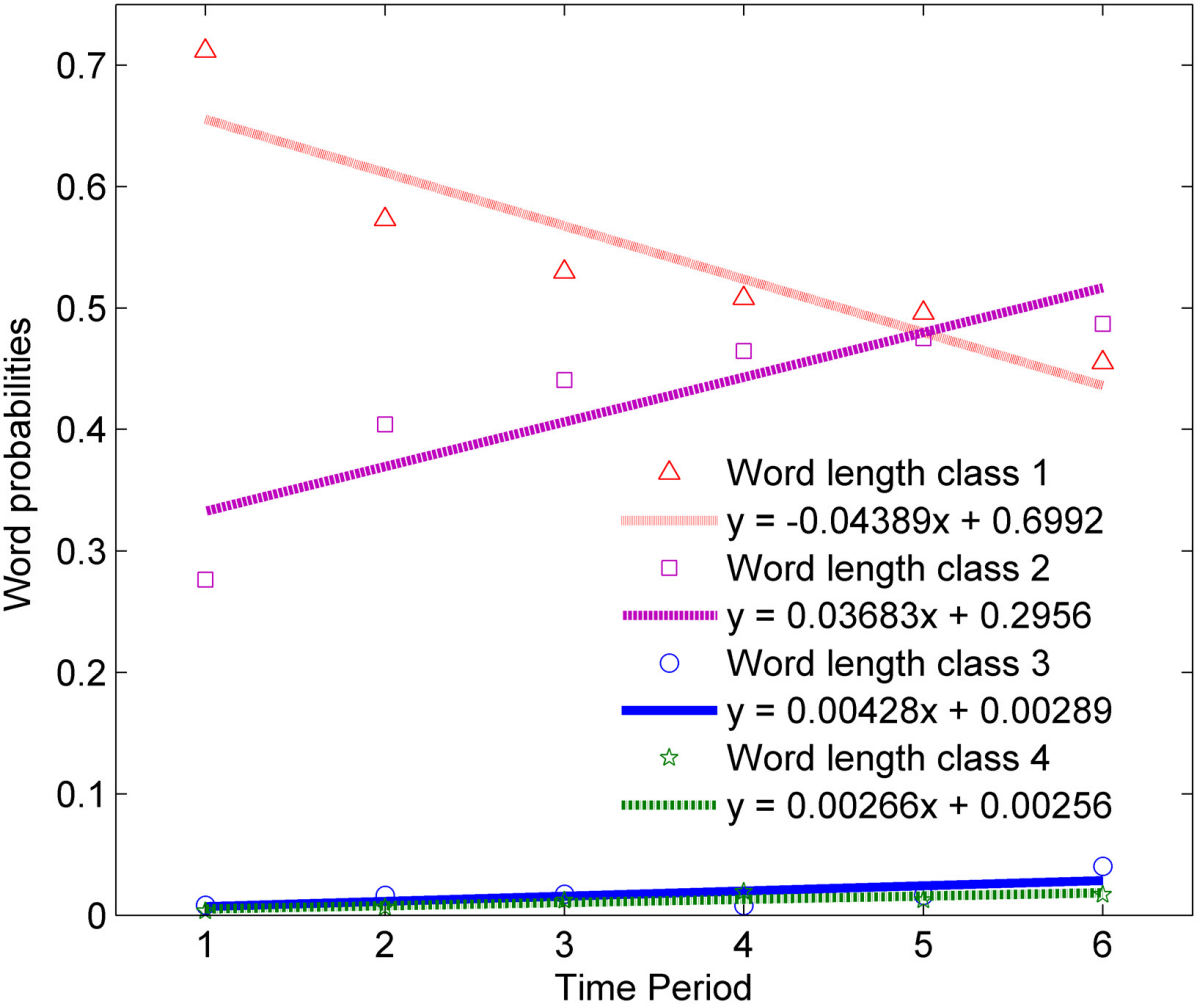
Linear fittings to static word probability changes of each word length class for texts with *N* = 1000.

To obtain a more accurate description of the evolution of each word length class, we also fit a linear model *y = ax+b* to the word probabilities of each word length class for texts of *N* = 1000. The fitting results are displayed in Fig 2.

Fig 2 shows the evolutionary speed of each word length class. It is about 800 years between Time Period (hence TP for short) 1 and 2, 2 and 3, 3 and 6. There are two jumps in the evolutionary process. One is from TP 1 to TP 2, the other is from TP 2 to TP 6. To test whether the word length distribution data from TP 1 to TP 2, and from TP 2 to TP 6 changed significantly, we adopted the Fisher’s Exact Tests. Results confirm our hypothesis with *p* = 0.001 and *p* = 0.002 respectively, suggesting that there is a fast growing probability in WLC 2, 3 and 4 from TP 1 to TP 2, as well as from TP 2 to TP 6. Further, it is in TP 6 that the probability of WLC 2 exceeds WLC 1 for *N* = 1000 texts.

Also, for the parameter *a* in the fitting function *y = ax+b*, only WLC 1 is negative, and the other three are positive. What is more, parameter *a* can be used as an indicator of the evolutionary speed. The larger the absolute value of *a*, the higher the evolutionary speed. Therefore, the evolutionary speed of WLC 1 is faster than the other three; compared with the WLC 1 and 2, the evolutionary speed of WLC 3 and 4 is much slower. Next we are probing into the dynamic one, i.e. dynamic word length distribution.

### Evolution of dynamic word length distribution

**Fig 3:**
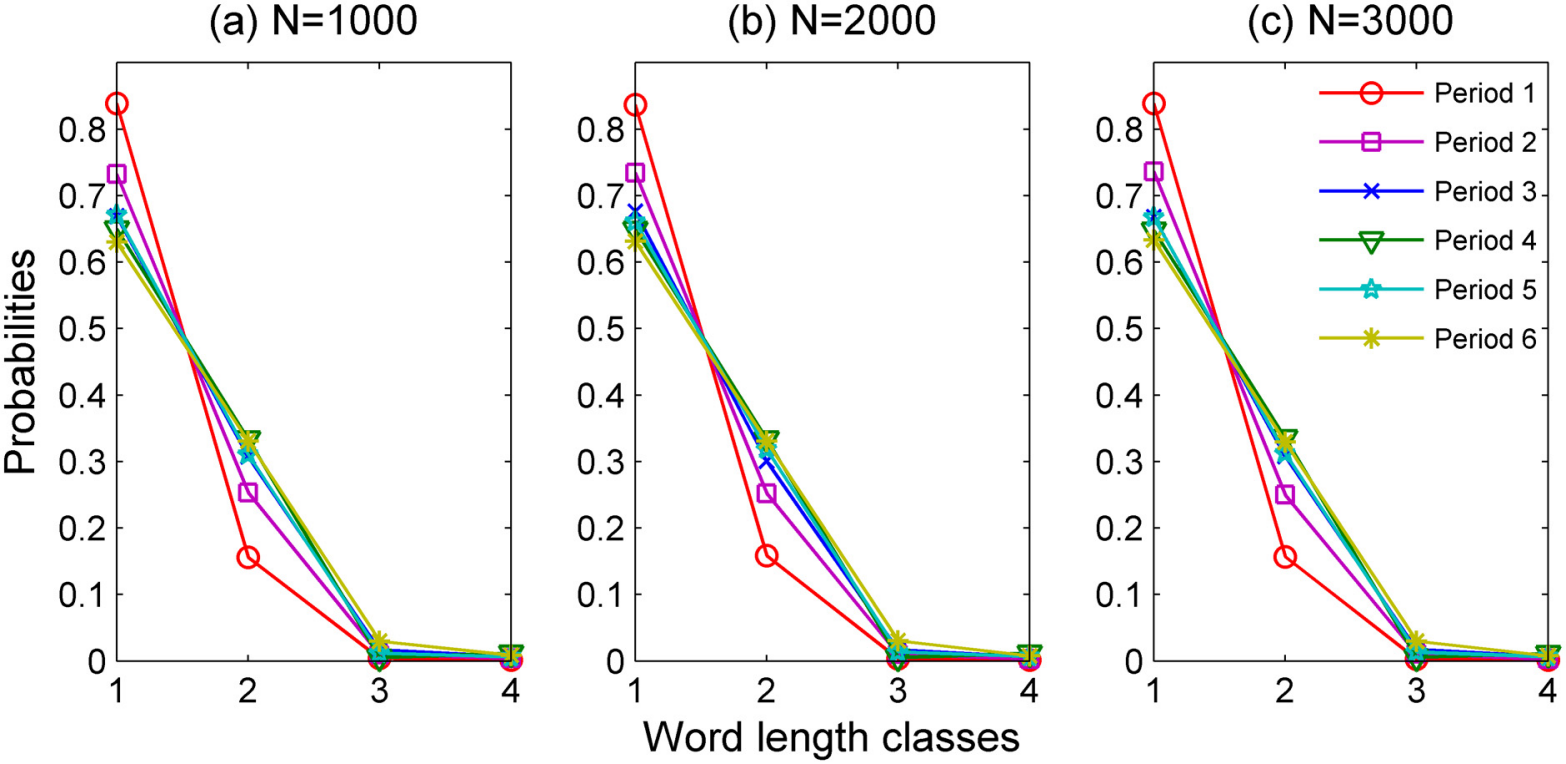
Dynamic Word length distributions of each time period with different texts scales.

Different from the static one, dynamic word length distribution reflects how static lexicons work in context of utterance in the principle of Zipf's "least effort". Fig 3 shows the diachronic and dynamic word length distributions (also with normalized words probability). Similar to Fig 1, Fig 3 shows an invariably decreasing trend in WLC 1, and a steady increasing trend in WLC 2, 3 and 4.

As can be seen in Fig 3 (see Table D in S1 File for the statistics), there is a dramatic drop in the probability of WLC 1: from 0.84 in TP 1 to 0.73 in TP 2. The other TPs have not witnessed manifested changes. Similar to the static word length distribution, the word probabilities in WLC 2, 3 and 4 increase over time.

However, different from Fig 1, Fig 3 clearly shows that WLC 1 has the highest word probabilities among different word length classes all the time. This phenomenon can be explained by Zipf's "principle of least effort", that is, speakers tend to use more short words in communications [11,12]. Nevertheless, the evolutionary trend of each word length class in Fig 3 is the same as in Fig 1. Now we are going to examine a common statistical index with regard to word length distribution, mean word length (hence MWL for short), to see if there are evolutionary regularities.

### Evolution of mean word length

MWL can be seen as an indicator and a simple estimate of lexical complexity of human languages. MWL is an important linguistic feature, and it is closely related with word length distributions. After observing a specific relation between the mean word length of a text and the relative frequencies of the individual word length classes, Čebanov was the first to suggest the Poisson distribution as a general word length distribution model for various languages [23].

There are two kinds of MWL, the dynamic one (DMWL) which refers to word tokens, and the static one (SMWL) which refers to word types. Similar to the differences between static and dynamic word length distribution, SMWL reflects the complexity of static lexicon, while DMWL reflects lexical complexity in dynamic context. See Materials and Methods for their algorithms.

**Fig 4:**
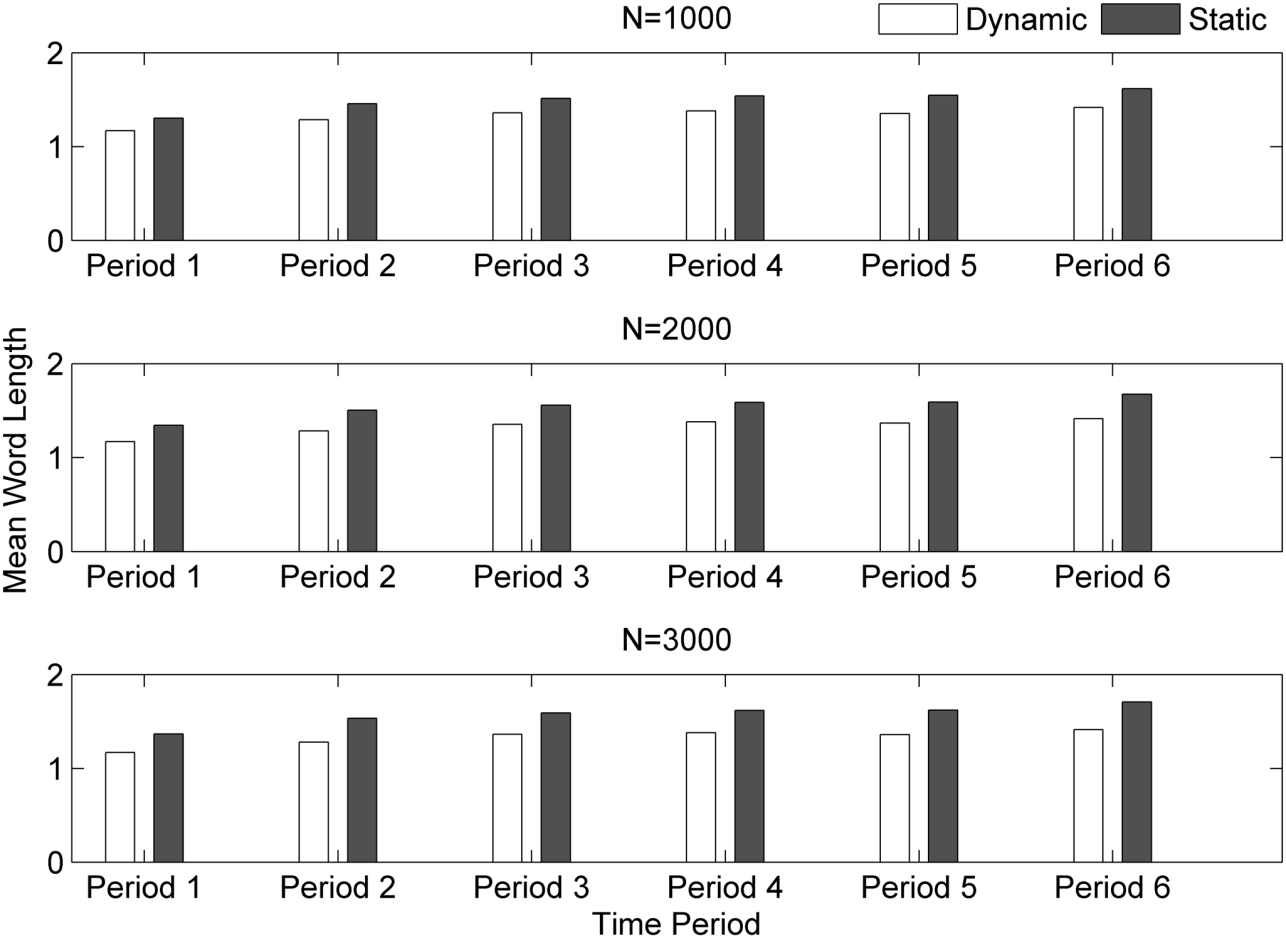
Dynamic and static mean word length evolution for different text scales.

The diachronic changes of DMWL and SMWL values are displayed in Fig 4 (Statistics for the sample in each TP can be found in S1 File).

MWL reflects the lexical complexity, or even linguistic complexity to some extent [25,26]. As we can see in Fig 4, both SMWL and DMWL increase with time in different text scales, which means that the lexical complexity of Chinese lexicon is increasing over time. To compare the evolutionary tempo of DMWL and SMWL, we fit a linear function *y = ax+b* to the data, and the values of *a* are 0.042 and 0.053 respectively, which means that DMWL evolves more slowly than SMWL. The mean word length is a general description about word length. To examine word usage of each word length class, we need to see another index, type-token ratio (hence TTR for short).

### Evolution of the relation between word length and type-token ratio

TTR reflects the usage of words in texts or contexts [12,21], and it can be calculated by the tokens of any one word length class divided by its word types. See Materials and Methods for their algorithms.

**Fig 5:**
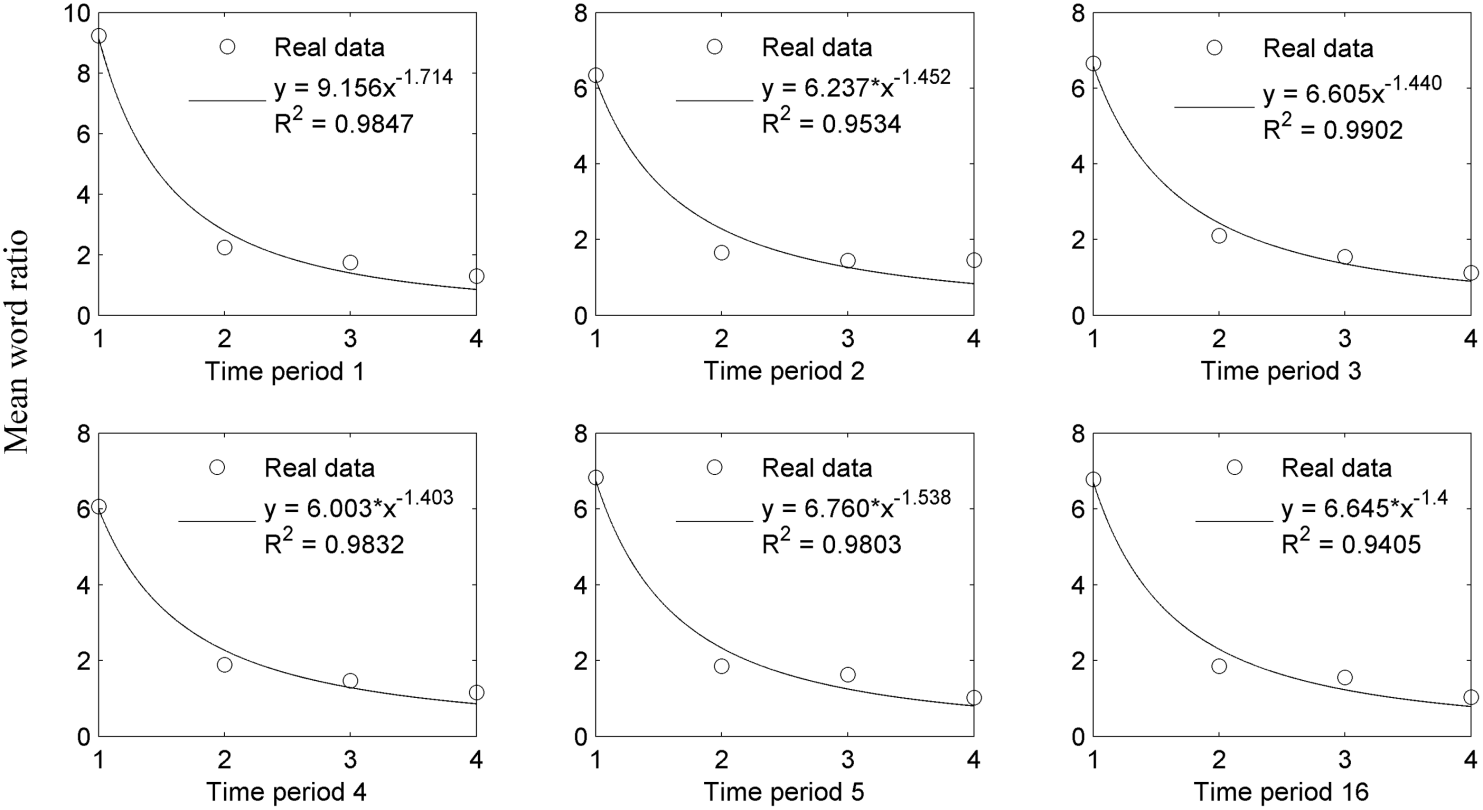
Fitting *y = ax-b* to the relation between word length and type-token ratio for each time period with *N* = 10000.

According to Zipf's claim that "the more frequent, the shorter" [9], here we put forward our hypothesis, "the shorter, the more frequent". We fit the Zipf's Power function *y = ax-b* to the relationship between word length and TTR. The fitting results are displayed in Fig 5.

As can be seen from the goodness of fit indicator *R2* in Fig 5, the data of all six time periods fit the Power law function *y = ax-b*. Hence, our hypothesis is consolidated that word frequency depends on word length throughout the evolution of Chinese word length. Zipf's law for word frequencies is a power law *y = ax-b*, with an exponent closely related with linguistic complexity. Baixeries et al.’s study shows that the values of the exponent *b* keep decreasing within the first 20 months of children, and then reach the level of adults [26]. Similarly, the values of the exponent *b* also shows a decreasing trend over time, as can be seen in Table G in S1 File (except in TP 5 that will be explained in **Discussions**). As the component *b* (in Fig 5) and SMWL (in Fig 4. We use it because it evolves more quickly than DMWL) both reflect the linguistic complexity, we believe that there is a correlation between them. Pearson Correlation analysis is employed to examine the relationship between *b* and SMWL, and a significant correlation coefficient is obtained at the 0.05 level (2-tailed): 0.894. This parallel evolution of the exponent *b* and a simple indicator of lexical complexity SMWL, supports that the exponent of the Zipf's law and linguistic complexity are inter-related and they both evolve over time.

## Discussion

Our results indicate that the increase of word length is an essential regularity of word evolution in written Chinese. These results perhaps can hold across languages for two main reasons. On the one hand, we focus on the diachronic aspects of language by examining the texts with a 2000-year spanning, which basically rules out the possible limitations of the widely used synchronic approaches. On the other hand, we take written Chinese texts for investigation. The classic written Chinese complements with modern written Chinese in that the former habitually uses mono-character words while the modern Chinese prefers to choose multi-character words to express the same meaning. We expect that this basic finding could generalize to other languages with a diversity of lexical systems.

In the evolution of static word length distributions, the probabilities of multi-character words and SMWL are increasing over time, and its distribution models are evolving towards a bell-shaped pattern, which is the general distribution pattern for many mature languages today [21–24]. Bochkarev et al. [27,28] claim that the changes of mean word length in a relatively short period of time can be influenced by socio-cultural factors. Our results suggest that the increase of mean word length can also be a primary result of word evolution in the long run. What is more, not only new concepts are coded in multi-characters, but also the old mono-character-words are recoded into multi-character-words, e.g. “邑” (*yì*) (which means capital in classic Chinese) is recoded into “国都” (*guódū*) (which means capital in modern Chinese).

In the evolution of dynamic word length distributions, there is an increasing use of multi-character words, and the values of DMWL also increase over time. Dynamic word length distribution is closely related with word frequency. In this study, the dependency of word frequency on word length is corroborated across all six time periods. To be specific, the relation between word length and TTR is in line with the power law function *y = ax-b*, which means that the shorter the word, the more frequent it is used. This indicates that word length and word frequency interact with each other in word evolution since word length can also be influenced by word frequency [10,12]. What is more, in the power law function *y = ax-b*, the value of exponent *b* increases over time (except in TP 5 that will be explained later), which means that although the power law works all the time, it evolves with the lexical system. The influence of the increase of word length is reflected in the exponent *b* changes: with the increase of multi-character words in the word inventory, their usage efficiency is enhanced too. Besides, the significant correlation between the values of exponent *b* and SMWL also confirms the word-length-increasing effect.

To our knowledge, this is the first attempt that explores how word length evolves in written Chinese from a quantitative point of view, and suggests that it is an essential element in the self-organizing lexical system. Admittedly, there have been many qualitative studies [29–32] concerning Chinese word evolution (to be specific, word formation). In this line of research, the relationship between spoken Chinese and written Chinese is a key point, and in light of their conclusions, we could ascertain that except TP 1 or even TP 2, the later TPs in our study should see a quite clear correspondence of one character in written Chinese to one syllable in spoken Chinese; and moreover consistent with their results [17–20,29], the disyllabic trend in Chinese word evolution is evident.

With regard to TP 1 or TP 2, previous studies show that, in Old Chinese (about from 1200 BC to 220 AD), there may be a sub-syllabic process, in which a light-syllable was followed by a full syllable and they eventually merged into one syllable [29,30,32]. As for the correspondence between character and its pronunciation in utterance, the reconstruction in Baxter and Sagart [30] shows that some characters may have a sesquisyllabic pronunciation. This means that, in TP1 or even TP 2, WLC 1 and 2 (WLC 3 and 4 account for only a very small portion of words which can be neglected in this case) in written Chinese may correspond to WLC 1, 1.5 and WLC 2, 2.5, 3 respectively in spoken Chinese in theory. However, even this situation is considered, the results may only be a decreasing portion of WLC 2 in TP 1 or even TP 2 (the original probabilities are 0.2764 and 0.4039 respectively), which means that the disyllabic trend may also be evident [29] (this can also be inferred from the increasing trend of WCL 2 as seen in Fig 2).

The above analyses show that the increasing portion of WLC 2 in written Chinese corresponds to the disyllabic trend in spoken Chinese, thus suggesting that the increase of word length could be an essential regularity in Chinese language. However, in TP 5, the modern Chinese Vernacular Movement in the early 20th century has changed this language to some extent, resulting in a simplified grammar and Europeanized sentences [33]. The simplification and Europeanization in Chinese grammar is one important reason why linguistic complexity and mean word length in TP 5 slightly decrease. As suggested by Bochkarev et al. [26], mean word length in a relatively short time period can be influenced by socio-cultural factors, and the modern Chinese Vernacular Movement accounts for this phenomenon.

Although there is a slight decrease of mean word length in TP 5, the increase of word length is the main trend in word evolution in written Chinese. As suggested by many studies, the primary reason may be that, as society develops, the increase of word length is inevitable because it is more efficient to express new meanings through combining signs rather than creating new signs [34,35]. This result corroborates the "error limit" theory put forward by Nowak et al., claiming that the increase of new concepts results in longer words [36].

The Chinese word length is increasing all the time within the last two thousand years, however, there exists a significant difference between Chinese and many Indo-European languages. On the one hand, in modern Chinese, almost over 80% Chinese words are mono-syllables (characters) or bi-syllables (characters), and the rest are rare. On the other hand, in other languages, such as English or German, there are more word length classes than in Chinese, and their distributions are closer to the bell-shaped pattern [21,23]. Here a question arises: why does the proportion of multi-syllable (character) words in Chinese lexicons evolve so slowly? The lexical development in Chinese language is a complex result of interactions between written and spoken Chinese [18,19,33]. As a kind of script that different from those of Indo-European ones, the Chinese character-combinations account for a lot of the new lexicons. We think it is probable that the evolution of Chinese language has also experienced the "error limit" effect, but the creation of more phonograms in written language increases the "error limit" to some extent, and hence hinders the development of multi-syllable (character) words in Chinese lexicon. As a result, the creation of more phonograms leads to more homophones.

On the whole, the increase of word length greatly adds redundancy to the lexical system (in both spoken and written language). Therefore, along with the increase of word length, the tone [37–40] and character [41] all become simplified for balance. The simplification of Chinese characters is systematic and continuous though there do exist some characters which become complex [19]. For example, the ancient character "漢"(*hàn*) is now simplified as "汉" (*hàn*). As for the simplification of tone, Peng [37] claims that some words or some syllables of certain words are getting fixed tones diachronically, e.g. high-rise, fall-rise, etc., which results in the reduction in tone types and weakening of distinction functions in tone. Similar views can be found in Wang [33]. Moreover, some studies even show that the phonological system (or syllable structure) is also simplified over time, which is accompanied by the disyllabic trend [29]. These changes in Chinese lexical system indicate that Zipf's "principle of least effort" works all the time in human communications.

Chinese is a different language type from Indo-European languages. However, the similarity lies in that they are both used for human communications. Human beings cannot create perfect words immediately. Wittgenstein compares language to a "game" [42], and a game must abide by some laws. We conclude that the increase of word length is an essential regularity in word evolution of written Chinese, which leads to a series of changes in the lexical system. To anticipate, this paper may shed some light onto the dynamics of word evolution. In the future research, we will explore the n-gram entropies of the diachronic Chinese texts in word length representation to test if the n-gram entropies are sensitive to the different time periods, and attempt to reveal the impact of word length correlations on the estimated n-gram entropies through comparison with the entropies of shuffled data.

## Materials and Methods

It is very important to choose the appropriate ancient texts, for there is an evident distinction between two kinds of written texts: the Literary Chinese and the Vernacular-style Chinese, of which the later stands much closer to the spoken language of that time, and the former is an independent system of written language that did not change too much since pre-Qin dynasty (about from B.C. 3 to Pre-A.D. 20). Such being the case, appropriate selection of diachronic language data is necessary for this study. Our diachronic study includes six historical periods (see Table A in S1 File). The texts of each time period are all short narrative texts of Vernacular-style Chinese which can represent the language used in that time [17,20,43].

A collection of stylistically homogeneous texts are also essential to this study. For this reason, firstly, we only select short narrative stories from one or two authors, as can be seen in Table A in S1 File; secondly, we further fragment the texts into segments of *N* = 1000 characters (generally speaking, samples of 1000 words are often used in quantitative investigations of languages such as in corpus linguistics or quantitative linguistics) [12,23]. The word length calculation is done over each one of these segments and the final value is obtained from averaging over all segments of each time period’s sample (ten thousand characters). At the same time, we also obtain the statics for the sample of each time period which can be seen in Tables in S1 File.

"Word" is difficult to define in Chinese for there is no space between words. For example,

Chinese: 词长是如何演化的?

Pinyin: Cí cháng shì rú hé yǎn huà de?

Chinese (after segmentation): 词长 是 如何 演化 的?

English (correspondence to words): (word length) (is) (how) (evolve) (particle ‘*de*’)?

English: How does word length evolve?

In the above Chinese sentence there are eight Chinese characters "词""长""是""如""何""演""化""的" and five words "词长""是""如何""演化""的". For there is no reliable word segmentation tools for ancient Chinese, we have to segment the ancient Chinese texts manually. To guarantee the impartiality of the results, the segmentation work was done by an expert in old Chinese. For the contemporary texts we use ICTCLAS 2008 [44] to segment words, and then an expert checked the segmentation manually and corrected the errors.

The chief problem of manual segmentation lies in the criteria for wordhood, especially when it comes to the distinction between words and phrases [45]. For the sake of consistency, we use the modern tokenization standard when we encounter these kinds of problems in manual segmentation, and refer to ancient dictionaries of that time to decide if the n-gram characters (mainly 2-gram characters) are words or not.

In Chinese, one syllable is roughly one character, and then word length can be calculated based on the number of characters in a word. In this paper we use word length class one to represent mono-syllable-words, and the like. For example, the word length of the five words in the Chinese sentence are "2""1""2""2""1" respectively.

After word segmentation, we developed our own java programs to obtain word length distribution frequencies and mean word length statistics. We used Matlab 2014a to obtain statistics and test if the relation between word length and type-token ratio fit the Power law function *y = ax* ^*-b*^. The functions for *DMWL*, *SMWL* and *TTR* are as follows.

Dynamic mean word length (*DMWL*) can be calculated with the following formula:

$$DMWL=\frac{\sum_{i=1}^{n}X_{i}F_{i}}{\sum_{i=1}^{n}F_{i}}$$

where *n* refers to the number of different word length classes, *X*_*i*_ refers to the word length of word length class *i*, and *F*_*i*_ refers to the word tokens of word length class *i*.

Static mean word length (*SMWL*) can be calculated with the following formula:

$$SMWL=\frac{\sum_{i=1}^{n}X_{i}F\prime_{i}}{\sum_{i=1}^{n}F\prime_{i}}$$

where *n* refers to the number of different word length classes, *X*_*i*_ refers to the word length of word length class *i*, and *F*_*i*_ *'* refers to the word types of word length class *i*.

And TTR can be calculated with the following formula:

$$TTR=\frac{F_{i}}{F\prime_{i}}$$

where *n* refers to the number of different word length classes, *F*_*i*_ refers to the word tokens of word length class *i*, and *F*_*i*_ *'* refers to the word types of word length class *i*.

## Supporting Information

S1 File Supporting Information. (DOCX)
