# Supplementary material for: How Does Word Length Evolve in Written Chinese?
Source: PLoS One. 2015 Sep 18;10(9):e0138567. doi: 10.1371/journal.pone.0138567 (PMC4575206; doi:10.1371/journal.pone.0138567)
Supplement: S1 File — (DOCX) [file pone.0138567.s001.docx]

**How does word length evolve in written Chinese?**

Supporting Information

Heng Chen^1^, Junying Liang^2^, Haitao Liu^2, 3*^

1. Center for the Study of Language and Cognition, Zhejiang University, Hangzhou, CN-310028, China. 2. Department of Linguistics, Zhejiang University, Hangzhou, CN-310058, China. 3. Ningbo Institute of Technology, Zhejiang University, Ningbo, CN-315100, China

1. Materials

Table A. Diachronic Corpus Details

| **Time** | **1** | **2** | **3** | **4** | **5** | **6** |
| --- | --- | --- | --- | --- | --- | --- |
| **Texts** | **Work** | **Work** | **Work** | **Work** | **Work** | **Work** |
|  | *MèngZǐ*  (Mencius) | *Shìshuōxīnyǔ*  (A New Account of the Tales of the World) | *Niǎn*  *Yùguānyīn*  (Grinding Jade Goddess of Mercy) | *Shíèrlóu*  (Twelve Floors) | *Nàhǎn*  (Yelling) | *Xīndàofózhī*  (The Buddha Knows Your Mind) |
|  | *Lǚshìchūnqīu*  (Mister Lv’s Spring and Autumn Annals) | *Yánshì*  *Jiāxùn*  *Shū*  (Mister Yan’s Family Motto) | *Cuòzhǎncuīníng*  (Wrongfully Accused of Ying Ning)  *Jiǎntiēhéshang*  (A letter from a monk) | *Wúshēngxì*  (A Silence Play) | *Pánghuáng*  (Hesitating) | *Huíménlǐ*  (A Wedding President) |
| **Scale**  **(characters)** | 141864 | 94729 | 11220 | 233430 | 91705 | 12980 |
| **Time**  **span** | B.C. 3th–  B.C. 2th | A.D.  4th –  A.D.  5th | A.D.  12th–  A.D.  13th | A.D. 16th–  A.D. 17th | Pre-A.D. 20th | A.D. 21th |

1. Methods

Dynamic mean word length (*DMWL*) can be calculated with the following formula:

|  | $DMWL=\frac{\sum_{i=1}^{n} X_{i}F_{i}}{\sum_{i=1}^{n} F_{i}}$ | (Equation A) |
| --- | --- | --- |

Where *n* refers to the number of different word length classes, *X_i_* refers to the word length of word length class *i*, and *F_i_* refers to the word tokens of word length class *i*.

Static mean word length (*SMWL*) can be calculated with the following formula:

|  | $SMWL=\frac{\sum_{i=1}^{n} X_{i}F'_{i}}{\sum_{i=1}^{n} F'_{i}}$ | (Equation B) |
| --- | --- | --- |

Where *n* refers to the number of different word length classes, *X_i_* refers to the word length of word length class *i*, and *F_i_'* refers to the word types of word length class *i*.

Type-token ratio (*TTR*) can be calculated with the following formula:

|  |  | (Equation C) |
| --- | --- | --- |

where *n* refers to the number of different word length classes, *F_i_* refers to the word tokens of word length class *i*, and *F_i_'* refers to the word types of word length class *i*.

3. Results (statistics)

3.1 Evolution of static word length distribution

Table B. Evolution of static word length distribution statistics for different text scales

| Text scale | Word length class | Time period | | | | | |
| --- | --- | --- | --- | --- | --- | --- | --- |
|  |  | 1 | 2 | 3 | 4 | 5 | 6 |
| N=1000 | 1 | 0.71185 | 0.57294 | 0.52983 | 0.50790 | 0.49597 | 0.45518 |
|  | 2 | 0.27644 | 0.40389 | 0.44048 | 0.46444 | 0.47510 | 0.48672 |
|  | 3 | 0.00837 | 0.01659 | 0.01743 | 0.00815 | 0.01641 | 0.04029 |
|  | 4 | 0.00335 | 0.00658 | 0.01226 | 0.01951 | 0.01252 | 0.01693 |
| N=2000 | 1 | 0.67575 | 0.52744 | 0.49055 | 0.46664 | 0.45613 | 0.41030 |
|  | 2 | 0.31041 | 0.44798 | 0.47636 | 0.50189 | 0.51073 | 0.52401 |
|  | 3 | 0.00976 | 0.01905 | 0.01875 | 0.00914 | 0.01873 | 0.04555 |
|  | 4 | 0.00407 | 0.00553 | 0.01434 | 0.02234 | 0.01441 | 0.01915 |
| N=3000 | 1 | 0.64950 | 0.49974 | 0.46285 | 0.44091 | 0.43098 | 0.38357 |
|  | 2 | 0.33902 | 0.47260 | 0.50183 | 0.52490 | 0.53287 | 0.54525 |
|  | 3 | 0.00682 | 0.02216 | 0.01908 | 0.00979 | 0.02036 | 0.04936 |
|  | 4 | 0.00465 | 0.00550 | 0.01624 | 0.02441 | 0.01579 | 0.02075 |

Table C. Evolution of static word length distribution for all the texts

| Word length class | Time period | | | | | |
| --- | --- | --- | --- | --- | --- | --- |
|  | 1 | 2 | 3 | 4 | 5 | 6 |
| 1 | 0.7167 | 0.5648 | 0.492 | 0.4714 | 0.4957 | 0.4442 |
| 2 | 0.2673 | 0.3964 | 0.452 | 0.484 | 0.4594 | 0.4666 |
| 3 | 0.0107 | 0.0228 | 0.0372 | 0.0114 | 0.0264 | 0.063 |
| 4 | 0.0053 | 0.016 | 0.0188 | 0.0332 | 0.018 | 0.024 |

3.2 Evolution of dynamic word length distribution

Table D. Evolution of dynamic word length distribution statistics for different text scales

| Text scale | Word length class | Time period | | | | | |
| --- | --- | --- | --- | --- | --- | --- | --- |
|  |  | 1 | 2 | 3 | 4 | 5 | 6 |
| N=1000 | 1 | 0.83848 | 0.73253 | 0.66930 | 0.64976 | 0.67100 | 0.63016 |
|  | 2 | 0.15590 | 0.25365 | 0.30744 | 0.33356 | 0.31087 | 0.33097 |
|  | 3 | 0.00410 | 0.00996 | 0.01687 | 0.00524 | 0.01191 | 0.02979 |
|  | 4 | 0.00152 | 0.00386 | 0.00639 | 0.01144 | 0.00623 | 0.00851 |
| N=2000 | 1 | 0.83676 | 0.73469 | 0.67618 | 0.64935 | 0.66086 | 0.63120 |
|  | 2 | 0.15848 | 0.25231 | 0.30081 | 0.33394 | 0.31882 | 0.33074 |
|  | 3 | 0.00322 | 0.01007 | 0.01678 | 0.00526 | 0.01366 | 0.03045 |
|  | 4 | 0.00154 | 0.00293 | 0.00623 | 0.01146 | 0.00666 | 0.00761 |
| N=3000 | 1 | 0.83855 | 0.73646 | 0.66820 | 0.64792 | 0.66755 | 0.63338 |
|  | 2 | 0.15700 | 0.25011 | 0.30784 | 0.33552 | 0.31257 | 0.32923 |
|  | 3 | 0.00273 | 0.01074 | 0.01741 | 0.00540 | 0.01342 | 0.02967 |
|  | 4 | 0.00171 | 0.00270 | 0.00656 | 0.01115 | 0.00645 | 0.00771 |

Table F. Evolution of dynamic word length distribution for all the texts

| Word length class | Time period | | | | | |
| --- | --- | --- | --- | --- | --- | --- |
|  | 1 | 2 | 3 | 4 | 5 | 6 |
| 1 | 0.7162 | 0.5648 | 0.492 | 0.4714 | 0.4957 | 0.4442 |
| 2 | 0.1333 | 0.1982 | 0.226 | 0.242 | 0.2297 | 0.2333 |
| 3 | 0.0035 | 0.0076 | 0.0124 | 0.0038 | 0.0088 | 0.021 |
| 4 | 0.0013 | 0.004 | 0.0047 | 0.0083 | 0.0045 | 0.006 |

3.3 Linear fitting results of the word probability changes of each word length class with *N*=1000.

Table 6. Linear fitting results of dynamic word length probability changes for each word length class with *N*=1000

|  | Word length classes | | | |
| --- | --- | --- | --- | --- |
|  | 1 | 2 | 3 | 4 |
| *a* | -0.03559 | 0.03066 | 0.00351 | 0.00135 |
| *b* | 0.82310 | 0.17480 | 0.00071 | 0.00161 |
| *R^2^* | 0.7539 | 0.7078 | 0.4812 | 0.5272 |

- 1. Mean word length evolution

Table G. Evolution of mean word length for different text scales

| Text scale | type | Time periods | | | | | |
| --- | --- | --- | --- | --- | --- | --- | --- |
|  |  | 1 | 2 | 3 | 4 | 5 | 6 |
| N=1000 | Static | 1.30321 | 1.45681 | 1.51212 | 1.53926 | 1.54548 | 1.61864 |
|  | Dynamic | 1.16866 | 1.28514 | 1.36036 | 1.37836 | 1.35336 | 1.41632 |
| N=2000 | Static | 1.34215 | 1.50267 | 1.55689 | 1.58718 | 1.59142 | 1.67322 |
|  | Dynamic | 1.16954 | 1.28123 | 1.35307 | 1.37884 | 1.36612 | 1.41448 |
| N=3000 | Static | 1.36663 | 1.53341 | 1.58871 | 1.61770 | 1.62096 | 1.70697 |
|  | Dynamic | 1.16760 | 1.27968 | 1.36233 | 1.37979 | 1.35877 | 1.41172 |

Table H. Evolution of mean word length for all the texts

| type | Time periods | | | | | |
| --- | --- | --- | --- | --- | --- | --- |
|  | 1 | 2 | 3 | 4 | 5 | 6 |
| Static | 1.47541 | 1.628861 | 1.680807 | 1.718591 | 1.71878 | 1.81392 |
| Dynamic | 1.168793 | 1.290989 | 1.360359 | 1.37836 | 1.353053 | 1.416324 |

3.5 Evolution of the relation between word length and type-token ratio

Table I. Evolution of the relation between word length and type-token ratio for N=10000

| Word length class | 1 | 2 | 3 | 4 | 5 | 6 |
| --- | --- | --- | --- | --- | --- | --- |
| 1 | 9.22938 | 6.34196 | 6.58635 | 6.06692 | 6.82782 | 6.78168 |
| 2 | 2.23283 | 1.66197 | 2.05829 | 1.88768 | 1.85093 | 1.85012 |
| 3 | 1.75000 | 1.45098 | 2.69565 | 1.46154 | 1.62963 | 1.55556 |
| 4 | 1.30000 | 1.45455 | 1.11905 | 1.15278 | 1.02273 | 1.03448 |
